# *USP44* Promoter Methylation in Plasma Cell-Free DNA in Prostate Cancer

**DOI:** 10.3390/cancers13184607

**Published:** 2021-09-14

**Authors:** Dora Londra, Sophia Mastoraki, Evangelos Bournakis, Martha Zavridou, Anastasios Thanos, Theodoros Rampias, Evi S. Lianidou

**Affiliations:** 1 Analysis of Circulating Tumor Cells, Lab of Analytical Chemistry, Department of Chemistry, National and Kapodistrian University of Athens, 15771 Athens, Greece; doralo@chem.uoa.gr (D.L.); smastoraki@chem.uoa.gr (S.M.); marthazavridou@hotmail.com (M.Z.); 2 Oncology Unit, 2nd Department of Surgery, Aretaieio Hospital, Medical School, National and Kapodistrian University of Athens, 11528 Athens, Greece; vagimith@yahoo.com; 3 Mutual Health Fund of National Bank of Greece Personnel, 11473 Athens, Greece; anastasios.thanos@gmail.com; 4 Basic Research Center, Biomedical Research Foundation of the Academy of Athens, 11527 Athens, Greece; trampias@bioacademy.gr

**Keywords:** cfDNA, liquid biopsy, real-time methylation specific PCR (MSP), *USP44*

## Abstract

**Simple Summary:**

Liquid biopsy provides real-time monitoring of tumor evolution and response to therapy through analysis of circulating tumor cells (CTCs) and plasma-circulating tumor DNA (ctDNA). *USP44* is a member of family proteins deubiquitinases, and plays an important role in cell growth; however, its accurate role in other cellular networks is under research. In this study, we examined for the first time *USP44* promoter methylation in plasma cell-free DNA (cfDNA) of patients with prostate cancer (early stage *n* = 32, metastatic *n* = 39) and 10 healthy donors (HD). *USP44* promoter methylation was detected in plasma cell-free DNA by a newly developed highly specific and sensitive real-time MSP method. We report for the first time that detection of *USP44* promoter methylation in plasma cell free DNA provides significant prognostic information in metastatic prostate cancer.

**Abstract:**

Liquid biopsy provides real-time monitoring of tumor evolution and response to therapy through analysis of circulating tumor cells (CTCs) and plasma-circulating tumor DNA (ctDNA). *USP44* is a critical gene which plays an important role in cell proliferation; however, its accurate role in other cellular networks is under research. *USP44* promoter methylation has been so far reported in colorectal neoplasia and metastatic breast cancer. In this study, we examined for the first time *USP44* promoter methylation in plasma cell-free DNA (cfDNA) of patients with prostate cancer (early stage *n* = 32, metastatic *n* = 39) and 10 healthy donors (HD). *USP44* promoter methylation was detected in plasma cell-free DNA by a newly developed highly specific and sensitive real-time MSP method. Our findings indicate that *USP44* promoter is methylated in plasma cell-free DNA of metastatic prostate cancer patients and that detection of *USP44* promoter methylation is significantly associated with overall survival (OS) (*p* = 0.008). We report for the first time that detection of *USP44* promoter methylation in plasma cell free DNA provides significant prognostic information in metastatic prostate cancer.

## 1. Introduction

Ubiquitination comprises a cellular post-translation modification, in which proteins can be modified through Lys-linked isopeptide bonds [1]. This signal leads proteins to their degradation, ubiquitin is attached to substrates by a sophisticated three-step enzymatic cascade utilizing E1 ubiquitin activating, E2 ubiquitin conjugating, and a variety of E3 ubiquitin ligating enzymes [2,3]. On the other hand, deubiquitinases can remove ubiquitin modification through the antagonism of E3 ligase and induce protein degradation [4]. *USP44* is a member of family proteins deubiquitinases, and plays an important role in cell growth. *USP44* regulates the separation of chromosomes in anaphase through deubiquitinasion of cdc20, a co-factor of *APC* gene [5,6]. Experiments in mouse models revealed that *USP44* modulated the mitotic checkpoint by regulating centrosome positioning and spindle geometry through a direct interaction with the centriole protein centrin. The function of *USP44* at the centrosome at least in part involves regulating the events surrounding centrosome separation, as its loss leads to an increase in cells with incomplete centrosome separation [7]. A second distinct tumor suppressive function of *USP44* is that it may play an important role in nucleotide excision repair (NER) by stabilizing *DDB2*; experiments in cell lines which were exposed to UVC radiation have shown that *DDB2*, a gene that plays role in NER mechanism, remains ubiquitin-free due to *USP44* function until XPC (Xeroderma pigmentosum) arrives and its activity further exists for subsequent rounds of damage recognition [8].

Epigenetic modifications consist of a genomic mechanism that influences gene expression without changes in the genome sequence [9]. In cancer, methylation plays an important role in many cellular pathways like apoptosis, differentiation, and cell growth [10]. DNA methylation is the first interaction between the genome and environmental factors [11]. *USP44* methylation is reported so far in a small number of studies in breast cancer, colorectal neoplasia, and non-small cell lung cancer [12,13,14]. RNA-sequencing (RNA-Seq) transcriptome data and DNA methylation data of the TCGA-BRCA revealed that USP44 methylation could be potential biomarker of breast cancer [15]. Experiments in breast cancer cell lines revealed that *USP44* hypermethylation promotes cell proliferation and metastasis [12].

Analysis of TCGA data in breast cancer has shown that *USP44* expression was significantly decreased when compared to normal [12]. In xenograft models and cells lines, *USP44* expression suppresses multipolarity and forms a bipolar spindle during mitosis. In KD *USP44* cells the multipolarity spindle pattern was induced, and the vasculogenic mimicry (VM) formation was inhibited, thereby suggesting that the planar-like and apico-basal–like spindle patterns promoted by *USP44* expression played a key role in VM, plasticity of aggressive cancer cells, and cancer metastasis [8]. *USP44* methylation is correlated with its low expression in tissue samples of colorectal neoplasia. The same results were observed in colorectal adenocarcinoma cell lines but the methylation of *USP44* did not correlate with aneuploidy [13]. Moreover, recent experiments in colorectal cancer using decitabine (a demethylating agent) has shown re-expression of *USP44* [13]. In contrast, according to the only study so far that reported on the *USP44* activity in prostate cancer, the expression of *USP44* appeared to promote tumor growth through the expression of the *EZH2* in prostate cancer cell lines, a histone-modifying enzyme which modulates the expression of genes involved in cell cycle and DNA repair. Immunoprecipitation assay in cell lines has shown an interaction between *USP44* and *EZH2* in catalytic activity. *USP44* KD cells have shown decreased levels of EZH2 protein [16].

Liquid biopsy, a minimally invasive blood-based testing, offers the possibility to follow-up tumor evolution in real time, and presents a significant approach that may soon change the management of cancer patients in diagnosis, prognosis, and therapeutic interventions. Liquid biopsy is based on the analysis of circulating tumor cells (CTCs), circulating tumor DNA (ctDNA), circulating miRNAs, and tumor-derived extracellular vesicles (EVs) that are shed from primary tumors and their metastatic sites into peripheral blood [17]. In clinical routine, multiple histological biopsies are required to monitor the response of cancer patients to treatment. However, tissue biopsies are invasive procedures and in some cases cannot be performed; for this reason, the scientific community has high expectations that liquid biopsy will surrogate tissue biopsies for the detection, prognosis, and therapeutic management of cancer patients [18,19,20].

In recent years, DNA methylation analysis in CTCs and ctDNA has shown strong potential, since it provides a valuable source of novel circulating epigenetic biomarkers for diagnosis, prognosis, risk assessment, and disease monitoring in many types of cancer [20]. Our group was the first to demonstrate epigenetic alterations in CTCs and corresponding ctDNA [21,22]. In breast cancer we reported that *ESR1* methylation in CTCs and ctDNA was correlated with lack of response to treatment [23,24]. Moreover, we reported that in breast cancer, *CST6* and *BRMS1* methylation was detected in ctDNA and CTCs, in ovarian cancer *RASSFIA* and *ESR1* methylation was detected in ctDNA, while in NSCLC *BRMS1* and *SOX17* methylation was detected in ctDNA [20].

In the present study we first investigated the molecular profile of prostate adenocarcinomas that are characterized by *USP44* promoter methylation through meta-analysis of TCGA data. We further evaluated for the first time the clinical significance of the detection of *USP44* methylation in plasma cell-free DNA of prostate cancer patients and we report a statistically significant association between *USP44* promoter methylation and overall survival.

## 2. Results

### 2.1. USP44 Promoter Methylation in Prostate Cancer

**Figure 1:**
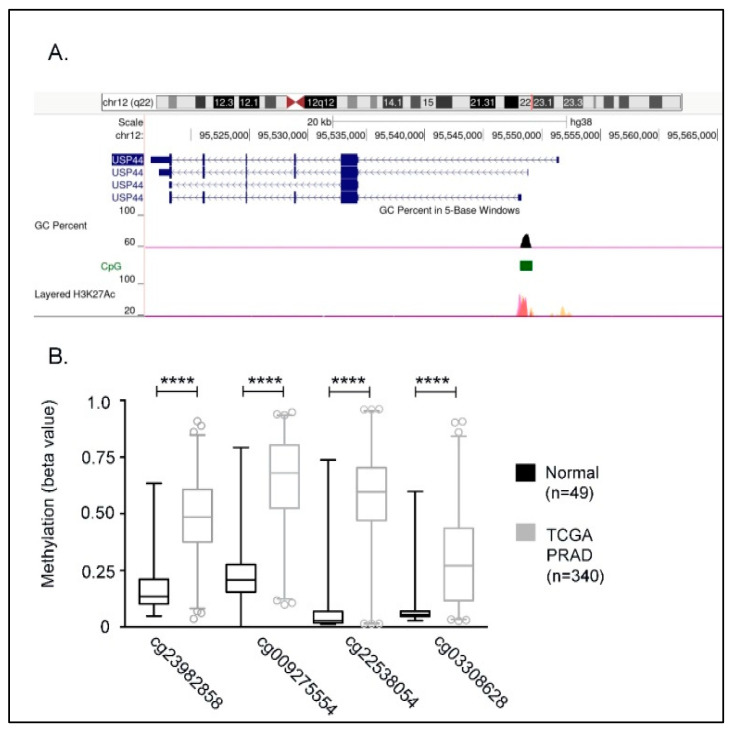
*USP44* promoter methylation. (**A**) Structure of *USP44* gene isoforms by ENCODE genome viewer. Promoter regions with high CpG context (black peaks) is indicated with green color. Transcriptional start site is indicated by peaks of H3K27Ac histone marks according to ENCODE data. (**B**) Cytosine sites within the indicated CpG island that display significant methylation in prostate adenocarcinoma samples as compared to normal samples. Methylation data were obtained from TCGA Wanderer database and Mann-Whitney test was used to analyze methylation levels between cancerous and normal samples. *p* values < 0.0001 are indicated with four asterisks (**** indicates *p* < 0.0001).

**Figure 2:**
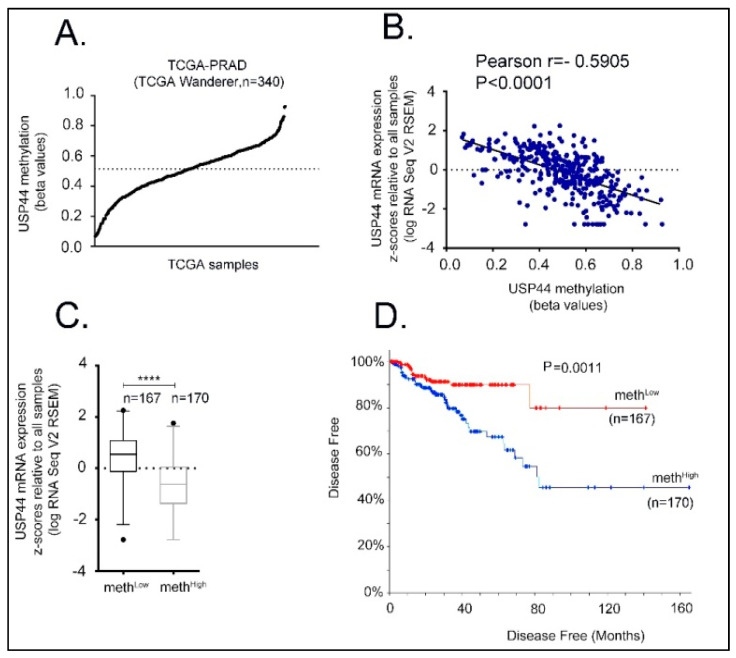
*USP44* promoter methylation and its correlation to prostate adenocarcinoma patient data. (**A**) *USP44* promoter methylation calculated by the average methylation values of cg23982858, cg009275554, cg22538054, and cg03308628 sites in TCGA prostate adenocarcinoma samples (TCGA-PRAD). Data were obtained from TCGA Wanderer database. Median *USP44* promoter methylation among TCGA samples is indicated by the horizontal line. (**B**) Correlation between *USP44* promoter methylation and mRNA expression. Expression data were obtained by TCGA-PRAD Firehose Legacy. (**C**) Boxplot of *USP44* mRNA levels in meth^High^ and meth^Low^ groups. *p* < 0.0001. (**D**) Disease-free curve comparing meth^High^ and meth^Low^ groups. Patient data were obtained by TCGA-PRAD Firehose Legacy through cbioportal. Log-rank test *p* = 0.0011 (**** indicates *p* < 0.0001).

According to ENCODE data, the proximal *USP44* promoter (hg19, chr1: 28735-29810) is characterized by the presence of a 1074 bp CG rich region (CpG 116, 73.2% GC content). This CpG island is enriched in H3K27Ac histone marks, indicating a critical role of this region in the transcriptional regulation of *USP44* locus (Figure 1A). To investigate whether prostate carcinogenesis is associated with DNA methylation in this CpG island, we first compared its methylation profile between prostate adenocarcinoma samples and normal prostate tissues using publicly available methylation data (TCGA-PRAD) obtained by the TCGA Wanderer database. As a result, we observed a higher level of methylation in prostate cancer tissues compared to that in normal prostate tissues (Figure 1B). More specifically, our analysis showed that among the Illumina methylation detection probes that are spanning the CpG island, the probes cg23982858, cg009275554, cg22538054, and cg03308628 display the higher difference on methylation (expressed as β values) compared to normal tissues. In a next step, the average methylation value of these probes was used to stratify the cohort of TCGA prostate adenocarcinoma samples with available methylation data (Figure 2A, TCGA-PRAD, TCGA Wanderer, *n* = 340). Based on the median value (β = 0.5144), the cohort samples were grouped either as meth^High^ (*n* = 170, β > 0.5144) or meth^Low^ (*n* = 167, β < 0.5144). Furthermore, we observed a negative association between *USP44* mRNA expression and the average methylation value of cg23982858, cg009275554, cg22538054, and cg03308628 probes in TCGA-PRAD tumors (Firehose Legacy)(Pearson r = −0.5905, *p* < 0.0001; Figure 2B). As expected, *USP44* mRNA levels were significantly lower in meth^High^ group of TCGA-PRAD tumors, compared to meth^Low^ group of tumors (*p* < 0.0001; Figure 2C). Notably, disease-free analysis of TCGA-PRAD (Firehose Legacy) patient data showed that disease free survival (DFS) was significantly shorter in meth^High^ compared to meth^Low^ group of patients (log-rank *p* = 0.0011; Figure 2D), indicating an association of *USP44* loss by methylation with aggressive disease.

### 2.2. USP44 Methylation and Its Correlation with Genomic Deletions That Affect the Molecular Properties of Prostate Adenocarcinomas

**Figure 3:**
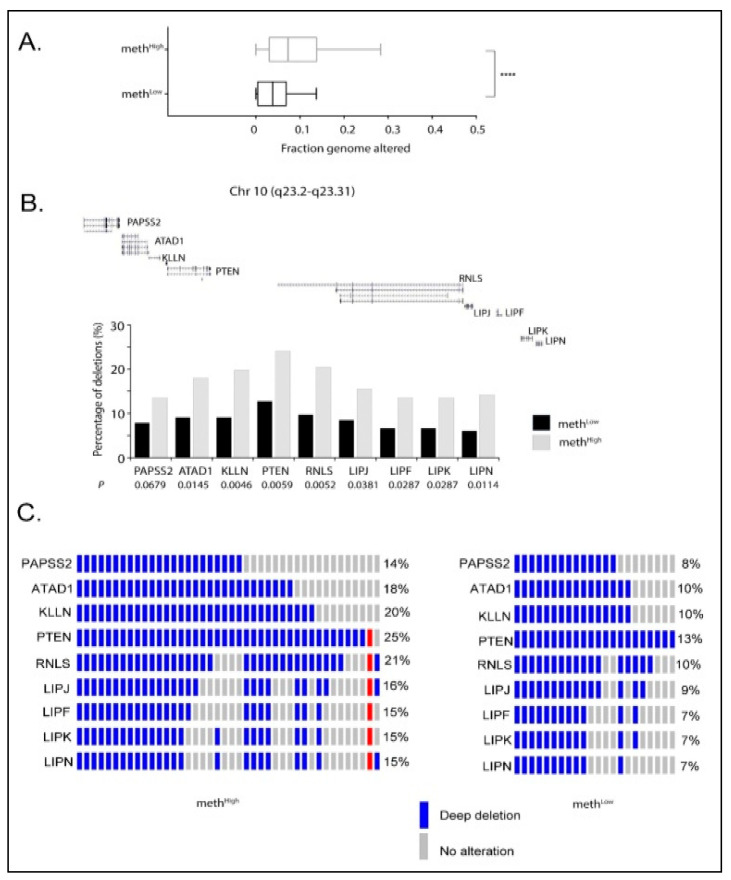
*USP44* methylation and its correlation to *PTEN* deletions in prostate adenocarcinoma patients. (**A**) Fraction of genome altered between meth^High^ and meth^Low^ groups. Data were obtained by TCGA-PRAD (Firehose Legacy). *p* > 0.0001. (**B**) *USP44* promoter methylation and correlation to gene deletions in chromosomal region Chr 10 (q23.2-q23.31). Copy number variation data (CNVs) were obtained by TCGA-PRAD (Firehose Legacy). *p* values underneath the gene names were calculated with Mann-Whitney test. (**C**) Oncoprints indicating patients with gene deletion events within the chromosomal region Chr 10 (q23.2-q23.31) in meth^High^ (left) and meth^Low^ (right) group. Gene deletion frequencies (%) within the group are indicated on the right side of the oncoprint. Oncoprints were generated through cbioportal using CNV data from TCGA-PRAD (Firehose Legacy).

The comparative analysis of copy number variation data for meth^High^ and meth^Low^ groups (TCGA-PRAD; Firehose Legacy) showed that prostate tumors with higher levels of *USP44* promoter methylation are characterized by a higher fraction of genome altered (*p* < 0.0001; Figure 3A). Moreover, this analysis revealed that loss of *USP44* expression by promoter methylation is highly correlated with deep deletions in the genomic region chr10q23.2-q23.3. As a result, the frequency of deletions in *PAPSS2*, *ATAD1*, *KLLN*, *PTEN*, *RNLS*, *LIPJ*, *LIPF*, *LIPK*, *LIPN* genes, which are located within this genomic region, is significantly higher in meth^High^ group compared to meth^Low^ group (Figure 3B). Notably, the tumor suppressor *PTEN* is deleted in 25% of meth^High^ and in 13% meth^Low^ group of tumors. As shown in Figure 3C, deletions of the tumor suppressor *PTEN* in meth^High^ tumors is associated with co-deletions of the other genes in close proximity within the chr 10q23.2-q23.3 region (*PAPSS2*, *ATAD1*, *KLLN*, *RNLS*, *LIPJ*, *LIPF*, *LIPK*, *LIPN*), indicating that this chromosomic region is sensitive to *USP44* loss associated genomic instability.

**Figure 4:**
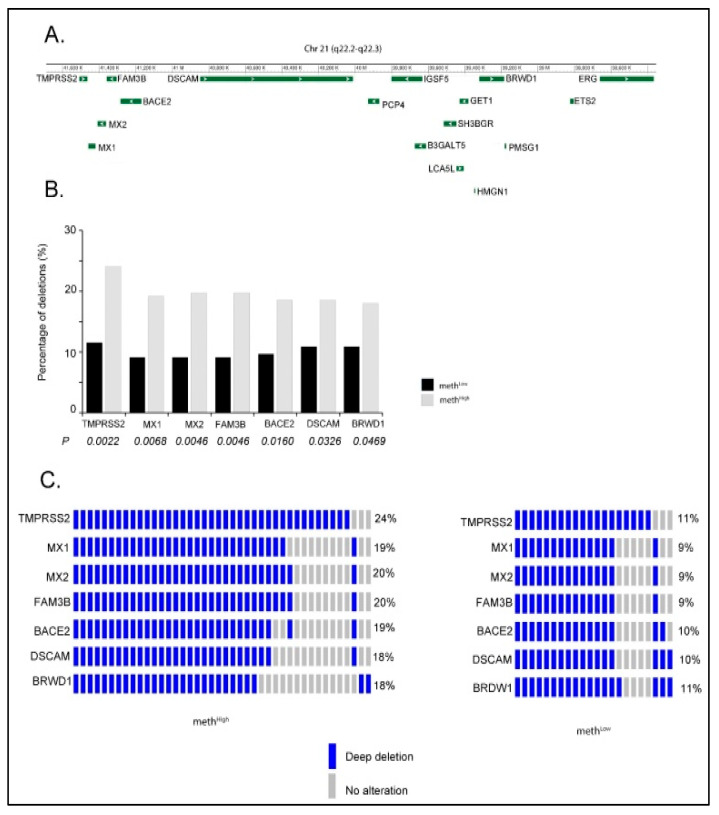
*USP44* promoter methylation and its correlation to deletions in the genomic region between *TMPRSS2* and *ERG* genes in prostate adenocarcinoma patients. (**A**) Genome view of the chromosomal region Chr 21 (q22.2-q22.3). (**B**) Copy number variation data for Chr 21 (q22.2-q22.3) for meth^High^ and meth^Low^ groups. CNV data were obtained by TCGA-PRAD (Firehose Legacy). *p* values underneath the gene names were calculated with Mann-Whitney test. (**C**) Oncoprints indicating patients with gene deletion events within the chromosomal region Chr 21 (q22.2-q22.3) in meth^High^ (left) and meth^Low^ (right) group. Gene deletion frequencies (%) within each group are indicated on the right side of the oncoprint. Oncoprints were generated through cbioportal using CNV data from TCGA-PRAD (Firehose Legacy).

Interestingly, the chr 21q22.2-22.3 genomic region (Figure 4A) was also identified to be associated with deep deletion events in prostate tumors with high levels of *USP44* promoter methylation. As a result, the frequency of deletions in *TMPRSS2*, *MX1*, *MX2*, *FAM3B*, *BACE2*, *DSCAM*, *BRWD1,* and *ERG* genes that are located within this region is significantly increased in meth^High^ compared to meth^Low^ group of tumors (Figure 4B). Moreover, as shown in Figure 4C, the copy number data indicate a profile of co-deletions for these genes in meth^High^ tumors, which is probably induced by the loss of the main body of the chr 21q22.2-22.3 genomic region in these samples. Notably, the deletion of the interstitial region between the *TMPRSS2* and *ERG* genes has been described as a mechanism for generation of *TMPRSS2-ERG* fusions (30).

### 2.3. Analytical Validation of the Developed MSP Assay for USP44

**Figure 5:**
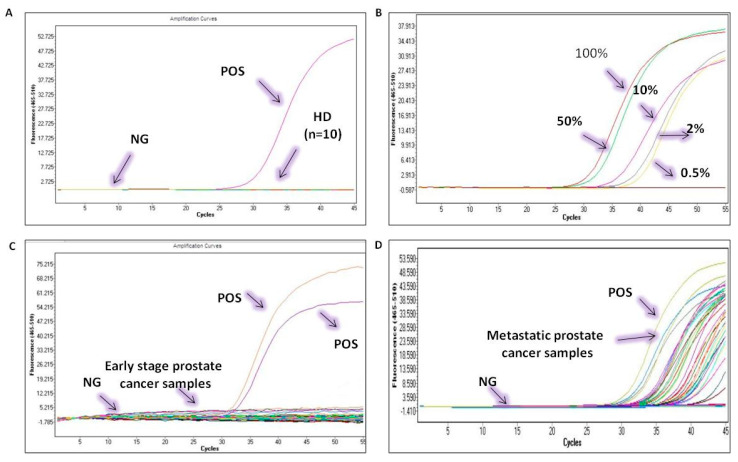
Analytical validation of the*USP44* promoter methylation real time MSP assay. (**A**) analytical specificity, (**B**) analytical sensitivity, (**C**) *USP44* RT-MSP in early-stage prostate cancer samples, (**D**) *USP44* RT-MSP in metastatic prostate cancer samples.

Analytical specificity: We initially evaluated the analytical specificity of the *USP44* real-time MSP, by testing the primers in silico and then in PCR, using SB-modified human placental gDNA samples that were not methylated; no amplification of the *USP44* was observed. In contrast, amplification was observed only when SB-treated DNA from the 100% methylated standard was used. The developed assay is highly specific since it detects only SB-treated *USP44* methylated sequences (Figure 5A).

Analytical sensitivity: The analytical sensitivity of the developed real-time MSP was evaluated by using synthetic mixtures based on serial dilutions of SB-converted DNA control samples (0% and 100% methylated) at various percentages of methylation (0.5%, 1%, 10%, and 50%). The developed real-time MSP assay for *USP44* methylation could specifically and reliably detect the presence of 1% methylated *USP44* sequences in the presence of 99% non-methylated *USP44* sequences (Figure 5B).

### 2.4. USP44 Methylation in Plasma Cell-Free DNA of Prostate Cancer Patients

The developed assay was first applied in 10 HD plasma samples to evaluate specificity; none of the HD plasma samples was found methylated at the *USP44* promoter (0/10, 0%) (Figure 5A).

We then examined the methylation status of *USP44* in plasma samples from 32 early prostate cancer patients; we did not observe methylation of *USP44* in this group (0%) (Figure 5C).

**Figure 6:**
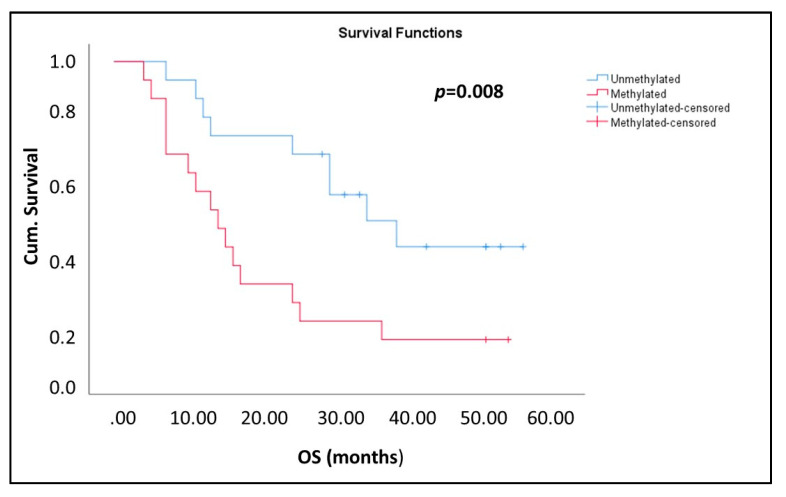
Kaplan–Meier estimates of patients with metastatic prostate cancer in relation to *USP44* methylation in ctDNA (*n* = 39).

We then examined the methylation status of *USP44* in plasma cfDNA samples from 39 prostate cancer patients with verified metastasis. In this group, 20/39 samples tested positive for *USP44* methylation (51.3%) (Figure 5D). Kaplan-Meier analysis has shown that in the group of metastatic prostate cancer patients, the detection of *USP44* methylation in plasma cfDNA was significantly correlated with worse OS (*p* = 0.008; Figure 6).

## 3. Discussion

In this study we report for the first time that detection of *USP44* promoter methylation in plasma cell free DNA provides significant prognostic information in metastatic prostate cancer, since it was significantly correlated with a worse overall survival. *USP44* is a member of family proteins deubiquitinases and plays an important role in cell growth. Targeted ubiquitination plays an essential role in the orderly progression of cells through mitosis. Our meta-analysis of TCGA prostate methylation and patient data indicate that high levels of *USP44* promoter methylation are associated with a more aggressive disease. *USP44* was recently found to play an important role in genome integrity maintenance, as it is an essential component of DNA damage response and mitotic checkpoint machinery [7,24,25,26]. Therefore, loss of *USP44* expression by methylation is expected to increase genomic instability in cancer. Towards this direction, our meta-analysis of TCGA copy variation data demonstrated that *USP44* promoter methylation is associated with deletions in genes that are located in chr10q23.2-q23.3 and chr 21q22.2-22.3 genomic regions including the *PTEN l*ocus and the interstitial region between *TMPRSS2* and *ERG* genes.

Numerous studies have shown that genomic deletion of *PTEN* is linked to prostate cancer recurrence and castration resistance [27,28,29,30]. On the other hand, several studies have demonstrated that deletion of the chr 21q22.2-22.3 genomic region and more specifically deletions of the interstitial genes between the androgen-regulated gene *TMPRSS2* and the *ETS* transcription factor family member *ERG* leads to the generation of a *TMPRSS2-ERG* fusion gene that promotes prostate cancer progression [31,32,33]. Therefore, the association of *USP44* promoter methylation with genomic alterations related to *PTEN* loss and *TMPRSS2-ERG* fusion formation provides further evidence for the prognostic value of this epigenetic alteration.

Few studies have attempted so far to elucidate the clinical significance of *USP44* promoter methylation in cancer patients. The first studies on *USP44* methylation were performed in mouse models and revealed that *USP44* loss caused spontaneous tumors (31). *USP44* expression analysis in human T-cell acute lymphoblastic leukemia (ALL) has shown overexpression of *USP44* compared to healthy donors (32). In vitro experiments in breast cancer and TCGA analysis suggested that *USP44* low expression was caused by promoter hypermethylation (12). The same group reported that *USP44* overexpression significantly suppressed cancer cell proliferation, migration, and invasion and induced apoptosis (12). In colorectal neoplasia an interaction between *USP44* promoter methylation and cancer was shown (13). Experiments in cell lines have shown that when *USP44* promoter was methylated, *USP44* mRNA transcripts were undetectable. It was also reported that *USP44* promoter was methylated in 89% of colorectal adenomas studied (*n* = 89), while in the matched normal colon mucosa samples only 3% (*n* = 51) were methylated. However, loss of *USP44* did not correlate with aneuploidy in colorectal adenomas (13). In NSCLC, there is only one study so far on *USP44* (14). In this study, *USP44* downregulation in 16 NSCLC tissues and in 4 cell lines was reported (14).

We evaluated for the first time the prognostic significance of *USP44* promoter methylation in plasma cfDNA from patients with prostate cancer. Towards this we firstly developed and analytically validated a novel highly specific and sensitive real-time MSP assay. Our findings indicate that in the early stage of the disease *USP44* promoter methylation was not detected in plasma cfDNA. On the contrary, in the metastatic setting, a high percentage (51.4%) of plasma cfDNA samples was positive for *USP44* promoter methylation, indicating that *USP44* promoter methylation may play an important role in this stage of prostate cancer. Moreover, according to our findings the detection of *USP44* promoter methylation was significantly associated with OS in these patients. So far, there is only one study on *USP44* in prostate cancer, based on the protein expression of this gene by immunofluorescence (33). According to the results presented in this study, *USP44* regulates enhancer of zeste homolog 2 (EZH2), a histone H3 lysine 27 methyltransferase, which is involved in the development and progression of various cancers (33). In vitro experiments performed in this study suggested that the loss of *USP44* reduced both the EZH2 protein levels and oncogenic activity of prostate cancer cells. Our results indicate on the contrary that loss of *USP44* through promoter methylation results in a worse OS in patients with metastatic disease. However, this could be explained by the fact that this study was based on prostate cancer cell lines and no clinical samples were used.

In conclusion, we report for the first time that *USP44* promoter is methylated at a high percentage in plasma cfDNA of metastatic prostate cancer patients but not in healthy donors and that detection of USP44 promoter methylation in plasma cell free DNA provides significant prognostic information in metastatic prostate cancer.

## 4. Materials and Methods

### 4.1. Prostate Adenocarcinoma TCGA Data and Statistical Analysis

All meta-analyses performed in this manuscript used data generated by The Cancer Genome Atlas Research (TCGA) Network and retrieved from cBioPortal (http://cbioportal.org (accessed on 20 April 2021) and TCGA Wanderer (http://maplab.imppc.org/wanderer/ (accessed on 25 April 2021) databases. Our analyses relied exclusively upon patient data which are publicly available. More specifically, methylation values (Illumina 450k Infinium chip data) from prostate tumor (*n* = 340) and normal samples (*n* = 49) were obtained from TCGA Wanderer. RNAseq, copy number variation, and clinical data for the prostate tumor samples with available methylation data were retrieved from the TCGA-PRAD Firehose legacy cohort.

Statistical differences in methylation (expressed as β values) of CG probes between normal and prostate tumors were determined by the non-parametric Mann-Whitney test. Pearson correlation coefficient was calculated to measure the linear relationship between USP44 mRNA expression and the mean methylation value of cg23982858, cg009275554, cg22538054, and cg03308628 sites among the TCGA samples. Based on the median value of methylation in these sites (β = 0.5144), the cohort samples were grouped either as meth^High^ (*n* = 170, β > 0.5144) or meth^Low^ (*n* = 167, β < 0.5144). The long-rank test was used to compare the disease-free survival between meth^High^ and meth^Low^ groups. Statistical significance of group enrichments in copy number alterations was accompanied by *p* values derived from chi-square test (*X*^2^). Statistical analyses were performed using GraphPadPrism ver.7.0. A *p* value of less than 0.05 was considered statistically significant.

### 4.2. Clinical Samples

Our study material consisted of a total of 71 clinical samples: (a) 32 plasma samples of patients with early stage prostate cancer; (b) 39 plasma samples of patients with metastatic prostate cancer from the second Department of Surgery at the Aretaieio University Hospital of Athens—Medical School, the National Kapodistrian University of Athens, and the Mutual Health Fund of National Bank of Greece Personnel; and (c) 10 plasma samples from healthy donors (HD). The study was conducted in accordance with the 1964 Declaration of Helsinki and was approved by the ethics and scientific committees of the participating institutions. All participating patients gave their signed informed consent in order to participate in the study.

### 4.3. Sample Preparation

To avoid contamination, different rooms, dedicated labware, and dedicated areas were used for all procedures. All DNA preparation and handling steps took place in specific laminar flow hoods under DNase-free conditions. DNA concentration in all cases was measured with a NanoDrop-1000 spectrophotometer (Thermo Scientific Waltham, Massachusetts, USA); isolated gDNA.

### 4.4. cfDNA Isolation from Plasma

Peripheral blood samples were collected into venous blood collection tubes using EDTA as a coagulant. Samples were mixed thoroughly and plasma was isolated within 2 to 4 h from sample collection by centrifugation at 530× *g* for 10 min at room temperature. Once isolated, plasma samples were centrifuged again at 2000× *g* for 10 min, before transferring into clean 2-mL tubes and freezing at −70 °C until time of processing. cfDNA was extracted from 2,00 mL plasma using the QIAamp^®^ Circulating Nucleic Acid kit 50 (Qiagen^®^, Hilden, Germany), according to the manufacturer’s instructions. Quality control checks were performed in every step before every experimental procedure. Cell free DNA integrity in all plasma samples was checked by amplifying a region in exon 20 of the *PIK3CA* gene as previously described [34].

### 4.5. Sodium Bisulfite Conversion

All samples with good quality of cell-free DNA (verified by positive amplification for *PIK3CA*) were further processed to Sodium Bisulfite (SB) treatment using the EZ DNA Methylation Gold Kit (ZYMO Research). Up to 500 ng cfDNA were used in each SB-reaction. The Universal Methylated Human DNA Standard (ZYMO Research) was used as fully methylated (100%) positive control. The quality of SB-treated DNA was checked by a real-time PCR assay for β-actin (*ACTB*). SB-converted DNA samples were stored at −70 °C until further use.

### 4.6. In Silico Design of MSP Primers

We first designed MSP primers for *USP44* in silico using the Primer Premier 5 software (Premier Biosoft International, San Francisco, CA, USA) avoiding the formation of stable hairpin structures, primer dimers, cross dimers, and false priming sites. The in silico validation was carried out using BLAST tool, in order to check their specificity and eliminate the risk of amplifying undesired sequences. The gene region of interest was selected by using the database TCGA Wanderer which provides gene expression and methylation data derived from TCGA database [35] (http://maplab.cat/wanderer (accessed on 20 April 2021)).

### 4.7. Optimization of Experimental Conditions

The experimental conditions of real-time MSP for *USP44* promoter methylation were first optimized in detail for the annealing temperature and time, then for the optimum concentrations of the primer pair, and finally for buffer, MgCl_2_, dNTPs, and BSA concentrations (data not shown). Each MSP reaction was performed in the 96-well plate LightCycler^®^ 480 System (IVD; Roche Molecular Diagnostics) (Mannheim, Germany) in a total volume of 10 μL. One microliter of SB-converted DNA was added to 9 μL reaction mixture containing 0.05 U·μL^−1^ GoTaq^®^ Hot Start Polymerase (Promega, Maddison, WI, USA), 0.2× of the supplied PCR buffer, 2 mM of MgCl_2_, 0.15 mM of each dNTP (Thermo Fisher Scientific, Waltham, Massachusetts, USA), 0.15 μg·μL^−1^ BSA, 0.2 mM of the forward and reverse primers, and 1× LC Green Plus Dye (Idaho Technology, Salt Lake City, UT, USA). Finally, deionized water was added to a final volume of 10 μL. Real-Time MSP protocol began with one cycle at 95 °C for 2 min followed by 45 cycles of 95 °C for 10 s, 65 °C for 20 s, and 72 °C for 20 s. Immediately after amplification, a rapid cooling cycle to 40 °C for 30 s was introduced in order to prepare the melting curve acquisition step. Real-time fluorescence acquisition was set at the elongation step (72 °C). The following melting curve analysis included the steps of 55 °C for 10 s, 92 °C for 0 s with a ramp rate 0.11 °C·s^−1^ (acquisition mode: continuous), 92 °C for 1 min, and 40 °C for 1 min.

## 5. Conclusions

In conclusion, we report for the first time that *USP44* promoter is methylated at a high percentage in plasma cfDNA of metastatic prostate cancer patients but not in healthy donors and that detection of *USP44* promoter methylation in plasma cell free DNA provides significant prognostic information in metastatic prostate cancer.

## Data Availability

The data presented in this study are available on request from the corresponding author. The data are not publicly available due to ethical restrictions.
